# Hernia

**Published:** 1828-11

**Authors:** 


### HERNIA.

### *Strangulated Scrotal Hernia. Removal of a Portion of Omentum.*

James Aldis, æt. sixty-eight, was admitted into the Middlesex Hospital on the 2d of August, labouring under strangulated scrotal hernia on the right side. He had had a rupture for many years, and had habitually worn a truss. Early in the morning of this day the rupture had come down in unusual volume, and had produced pain in the abdomen. The tumor was hard and tense. The usual remedies were tried; a large injection was given; the patient was placed in the warm bath, and bled so as to produce faintness; and, while he was in this state, continued attempts were made, which lasted nearly an hour, to reduce the hernia by the taxis. The tumor, however, did not yield to these attempts; nor was it rendered less tense, but it became more painful. Under these circumstances it was thought right not to delay the performance of the operation.

The contents of the sac were found to be a portion of the colon, and a quantity of indurated omentum. The intestine was speedily returned; but the mass of condensed and thickened omentum did not admit of being reduced. Mr. Mayo, therefore, cut it off close upon the ring, and (having tied as many as ten arteries, each with a single thread,) returned what remained into the abdomen.

This patient for several days was in great danger, with a dry tongue, and a pulse varying from 120 to 140 in a minute, attended with delirium. There was pain and tenderness of the abdomen during the first two days after the operation: the latter symptom now, and on its recurrence four days afterwards, was removed by means of leeches and fomentations. After the operation, when the bowels had been freely opened, the patient continued, for several days, to take a grain of calomel, and two of pulvis antimonialis, every eight hours. Under this treatment he gradually recovered.

---

*Strangulated Inguinal Hernia. Intestine opened.*

John Quick, æt. forty-two, was admitted into the Middlesex Hospital, October 2d, with strangulated inguinal hernia in the right side. The rupture was congenital. He had constantly worn a truss for many years, and when it happened that the hernia came down he had found no difficulty in replacing it. On the present occasion, his bowels had been confined for several days, and the rupture had come down the preceding evening, while he was engaged in no unusual bodily exertion. He was bled during the night of the 1st of October, and ineffectual efforts were made to reduce the hernia.

When Mr. Mayo saw this patient, soon after his admission into the hospital on the morning of the 2d, the tumor was tense, and tender on pressure; there was tenderness of the abdomen, nausea, (he had vomited once or twice,) a sense of dragging from the epigastrium, hiccup, an anxious countenance, and an irregular pulse. A large injection was given, which speedily operated. The patient was placed in a warm bath, and attempts were cautiously made to reduce the hernia; but the patient complained of so much pain that they were discontinued, and the operation was performed.

The sac was found to contain about a foot of small intestine, which was dark, highly inflamed, and pretty firmly adherent to the neck of the sac, and here and there so discoloured as to shew that gangrene was commencing.

The adhesion of the intestine to the neck of the sac being broken through with the finger, the stricture divided, and the intestine opened, the aperture was made fast to the divided integuments by a ligature, and the rest of the bowel returned into the belly. After the operation, the pulse became regular, the bowels acted freely through the wound as well as by the rectum, and the pain and tenderness of the belly diminished. The latter symptoms were found in some degree increased the following day, when he was bled, and since then his progress towards recovery has been uniform.

---

